# 以溶血性贫血及血小板减少为首发临床表现的儿童谷固醇血症1例报告并文献复习

**DOI:** 10.3760/cma.j.cn121090-20230915-0068

**Published:** 2024-01

**Authors:** 子悦 赵, 津婴 李, 韦华 黄, 丽玲 邱, 宝华 钱, 占山 查

**Affiliations:** 海军军医大学第一附属医院，全军儿童溶血性贫血研究创新基地，上海 200433 Department of Blood Transfusion, Changhai Hospital, The First Affiliated Hospital of PLA Naval Medical University, Research &Innovation Base of Pediatric Hemolytic Anemia, Shanghai 200433, China

## Abstract

报道1例表现为溶血性贫血和血小板减少的儿童谷固醇血症病例。谷固醇血症是一种罕见的常染色体隐性脂质代谢障碍，由于其非典型临床表现，诊断具有挑战性。研究强调了识别此病的重要性，尤其是在表现为溶血性贫血和血小板减少的患者中。案例涉及1例最初被误诊为丙酮酸激酶缺乏的8岁儿童。进行了详细的生化和分子分析，包括基因测序。结果显示ABCG5基因的纯合突变，确诊为谷固醇血症。这一病例强调了需要综合诊断方法和提高临床的认识。通过分析1例儿童病例，展示了谷固醇血症的复杂性和诊断难度。这名8岁儿童最初被误诊为丙酮酸激酶缺乏症，后经过全面的生化和分子生物学分析，包括基因测序，最终确诊为谷固醇血症。该病例表明，对于表现为溶血性贫血和血小板减少的患者，医生需要考虑更广泛的诊断可能性，以减少误诊。这一研究强调了对谷固醇血症的认识和准确诊断的重要性。

## Abstract (en)

This article focuses on a case study of sitosterolemia in a child who initially presented with hemolytic anemia and thrombocytopenia. Sitosterolemia is a rare autosomal recessive lipid metabolism disorder, difficult to diagnose due to its non-typical clinical manifestations. The 8-year-old patient was initially misdiagnosed with pyruvate kinase deficiency. Comprehensive biochemical and molecular biology analyses, including gene sequencing, eventually led to the correct diagnosis of sitosterolemia. This case highlights the complexity and diagnostic challenges of sitosterolemia, emphasizing the need for increased awareness and accurate diagnosis in patients presenting with similar symptoms.

谷固醇血症（STSL）又称谷甾醇血症或植物固醇血症，是一种罕见的常染色体隐性遗传的脂质代谢疾病，由Bhattacharyya和Connor在1974年首次描述[1]。其发病的分子基础是三磷酸腺苷结合盒G亚家族（ABCG）5和ABCG8编码基因突变后植物固醇经肠道吸收增加，经胆汁排泄减少，导致血清中植物固醇（如β-谷固醇、油菜固醇、豆固醇）显著升高[2]–[4]。STSL最重要的临床表现包括肌腱黄色瘤、皮肤结节性黄色瘤、早期关节痛、关节炎和动脉粥样硬化；血液学异常通常与其他临床症状一起出现，但偶尔也可能是唯一的临床症状。主要相关的血液学异常包括溶血性贫血、口形红细胞增多、血小板减少、大血小板和脾肿大[5]–[6]。溶血性贫血和血小板减少是临床常见的血液学异常，大多数医师较难认识到其或与STSL相关[7]–[8]。因此，STSL的临床诊断困难，误诊率高。由于STSL的临床表现的不典型性和临床诊断的特殊性，临床医师需提高对该病的认识。现报道1例以溶血性贫血及血小板减少为首发临床表现的病例如下。

## 病例资料

患儿，男，8岁，祖籍四川。患儿3岁时因“发热”就诊，血常规检查发现中度贫血，给予“补血药物”口服治疗半个月，无明显效果，后随访HGB仍低，家属未行进一步诊治。2020年12月为明确贫血原因携患儿再次就诊，以“中度贫血待查”收住院。腹部彩超示：肝脾大。骨髓涂片示：骨髓有核细胞增生明显活跃，红系增生明显，成熟红细胞大小不一；巨核细胞易见，功能尚可。考虑地中海贫血可能性大，进一步检测地中海贫血基因，基因检测未见致病突变。此后未再进一步诊治。2023年1月因“发热、咳嗽”再次入院，血常规示RBC 1.88×10^12^/L、HGB 57 g/L、网织红细胞比值（Hct）20.5％、平均红细胞体积（MCV）109.0 fl、平均血红蛋白含量（MCH）30.0 pg、平均血红蛋白浓度（MCHC）278 g/L、PLT 50×10^9^/L、血小板体积分布宽度（PDW）34.9 fl、平均血小板体积（MPV）20.6 fl、大血小板比率（P-LCR）110.8％。叶酸4.88 nmol/L、铁蛋白169.7 ng/ml、维生素B_12_ 637.2 pmol/L。B超显示：胆胰肾形态大小正常；肝大，形态饱满，剑突下50 mm，肋缘下50 mm，肝右叶最大斜径约120 mm；脾大（163 mm×60 mm），肋缘下55 mm，脾周围见一中等回声团，大小约14 mm×11 mm，考虑副脾。血脂正常。直接抗人球蛋白阴性，间接抗人球蛋白阴性。凝血功能检测正常。人细小病毒B19抗体检测阴性，传染病八项阴性。对患儿血液样本进行红系相关遗传性疾病基因检测。二代基因测序报告一种可疑变异——PKLR杂合突变（千人组基因频率0.004 36），c.1516G>A（p.V506I），拟诊断为丙酮酸激酶（Pyruvate Kinase，PK）缺乏症。

为明确患儿是否为PK缺乏症，故进行溶血病相关检测：①血涂片：红细胞体积大小不一，浅染、部分细胞中央淡染区缩小，口形细胞大于20％，可见巨大血小板（图1）。②血红蛋白电泳：未见异常区带，HbA2 3.6％、HbF 1.0％、HbA 95.4％。③异丙醇实验阴性。④醋酸纤维素膜电泳阴性。⑤MCV/RBC比值排除地中海贫血。⑥酸化甘油实验：AGLT50大于290 s，正常。以ICSH推荐的速率法检测红细胞酶病，结果显示患儿丙酮酸激酶27.44 IU/g Hb［正常参考（14.44±1.22）IU/g Hb］，G6PD、P5′N正常。⑦患儿父亲PK活性为16.38，患儿母亲PK活性为17.43，均未见酶活力缺乏。

**图1:**
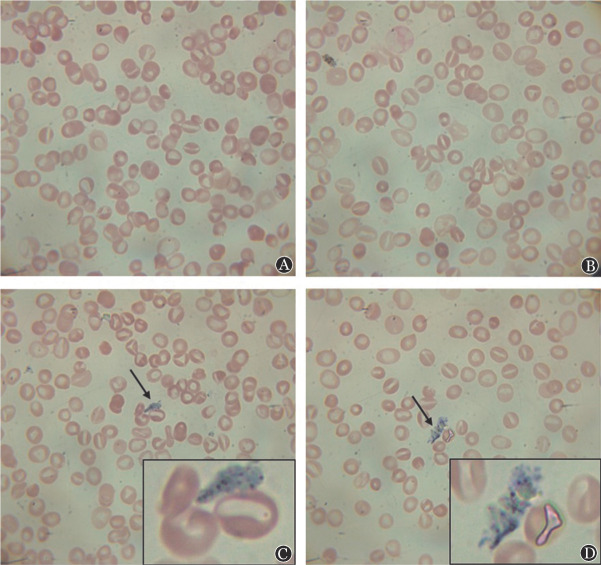
谷固醇血症患者外周血瑞氏染色红细胞和血小板形态 **A** 红细胞形态双向改变；**B** 口形红细胞；**C**、**D** 巨大血小板

本室检测未见酶活力缺乏，排除隐性遗传PKLR杂合突变导致溶血的可能性。由于镜下观察外周血涂片，可见较多的口形红细胞，故检索基因测序原始数据中与口形相关的膜蛋白基因KCNN4、SLC4A1、PIEZO1、RhAG、SLC2A1、ABCB6。结果显示虽然存在基因多态性改变，但多态性概率在0.049～0.97之间，在千人基因组（1000 g 2015 aug_ALL）数据库中，均为良性变异，可排除遗传性口形红细胞增多症。由于本案例患儿有骨痛、关节痛的临床表现，红细胞形态为口形变化、血小板为大体积改变，Coombs试验阴性，合并基因测序检索结果，排除血栓性血小板减少性紫癜、戈谢病、尼曼匹克等疾病的可能，高度提示患儿可能为STSL。再次检索基因检测结果，发现该患儿ABCG5基因有1个纯合突变（千人组基因频率0.000 87），ABCG5基因第10外显子编码区第1336号核苷酸由胞嘧啶变异为胸腺嘧啶，导致第446位精氨酸发生无义突变（c.1336C>T，p.R446X）（图2），该变异在人群中发生频率极低，与STSL相关。在该患儿样本中尚未发现ABCG8基因的突变。经家系验证分析，受检患儿的父亲和母亲在该位点均存在杂合变异（图2B、C）。结果表明，该患儿系ABCG5纯合突变导致的以溶血和血小板减少为主要临床表现的STSL。

**图2:**
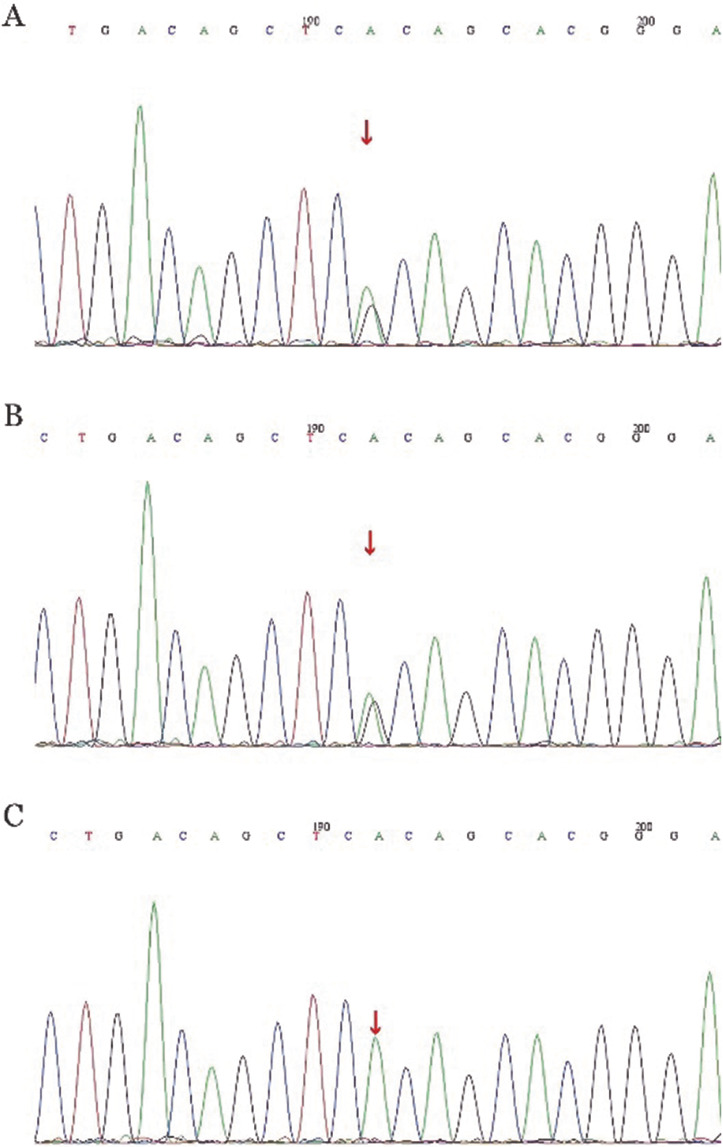
谷固醇血症患儿及父母ABCG5基因部分核苷酸序列（箭头所示为突变位点） **A** 患儿；**B** 患儿父亲；**C** 患儿母亲

## 讨论及文献复习

ABCG5/ABCG8引起的STSL是一种罕见的常染色体隐性遗传病，它会导致人体内谷固醇和其他植物固醇的积累，同时降低胆固醇水平[9]–[10]。该病的临床特点比较多样化，主要包括如下方面：①脂质代谢异常[11]–[12]：患者的血液中，会出现植物谷固醇、菜油固醇和豆固醇等不同类型的固醇升高，而胆固醇和三酰甘油则降低，这可能导致肝功能异常；②消化系统表现[13]：ABCG5/ABCG8基因的突变会影响胆汁酸的合成和排泄，导致胆道结石、胆汁淤积等问题。患者常常会有腹痛、腹泻、黄疸等症状；③皮肤表现[14]：患者可能有黄色小疙瘩（黄色瘤）的皮肤病变；④心血管疾病[15]：植物固醇积累可能增加冠心病和动脉粥样硬化的发病风险；⑤血液系统疾病[7]：患者可能会出现贫血、口型红细胞增多、巨大血小板伴血小板减少、脾大。总之，ABCG5/ABCG8引起的STSL是一种单系统或者多系统受累，临床表现复杂的疾病。对于存在相关症状或家族史的患者，建议及时进行基因测序以便早期诊断和治疗[16]。

在正常情况下，人类血浆植物固醇水平低于10 mg/L，仅占血浆固醇总含量的0.2％。ABCG5和ABCG8的突变导致血浆中植物固醇的浓度升高30～100倍，而血浆胆固醇水平正常或轻度至中度升高[17]。本例患儿的胆固醇及其他血脂类指标均在正常范围内，这可能是一直未表现出皮肤黄色瘤、动脉粥样硬化的原因。但是该患儿出现的血液学异常，这可能是植物固醇在血细胞细胞膜中蓄积，改变细胞膜硬度，使细胞膜容易破裂，并导致形态和功能异常[18]；另一些作者还发现了在STSL患者的体内血小板过度活化、αIIbβ3表达减少、GPIba-FlnA连锁缺失和微颗粒形成，因此，植物固醇在血小板膜中的富集可能影响血小板的大小、数量和功能[19]。Wang等[20]报告了13例STSL，其中2例患者口形红细胞增多和巨大血小板伴血小板减少的溶血性贫血是唯一临床表现。当溶血性贫血和血小板减少症并存时，大多数医师首先想到是遗传性溶血性贫血、血小板减少性紫癜、巨大血小板综合征、Evans综合征或自身免疫性疾病[18],[21]。一般来说，口形红细胞增多和巨大血小板伴血小板减少的共存是少见的，仅在STSL和骨髓增生异常综合征中报告。应该强调的是，仔细检查外周血涂片是STSL诊断方法的最重要方面[22]–[23]。但同时也应排除口形相关基因的突变。

血液学异常为首发症状的STSL的诊断要点：主要观察血象、红细胞形态、临床指征和基因突变四方面表现。血常规表现贫血、血小板减少伴巨大血小板，注意PDW、MPV、P-LCR均为增大改变。血细胞形态上，可见口形红细胞增多伴大体积血小板。在临床表型上，存在血管外溶血指征、脾大、关节痛、骨痛，有些病例可见黄色瘤和动脉粥样硬化。基因突变特征：ABCG5、ABCG8基因纯合突变可导致STSL。

STSL的患者需要长期接受治疗。虽然目前尚无特效治疗方法，但是通过饮食控制、药物治疗等手段可以有效地缓解该疾病的症状和进展。饮食方面，碳水：推荐大米、马铃薯和甘薯；脂类：建议用猪油或者牛油代替植物油；蛋白质类：减少贝类和海藻类植物蛋白摄入，因其含有大量藻类衍生的植物固醇，还有黄豆、青豆和黑豆中植物固醇的含量相对较高，都应减少摄入[24]。药物治疗方面，依折麦布和胆汁酸封存剂可以进一步降低血浆中植物固醇的浓度[3]。也有文章表明依折麦布有利于增加血小板计数[15]。本文患儿在10 mg/d依折麦布口服半年后，复查血常规发现PLT由50×10^9^/L上升到103×10^9^/L，HGB由57 g/L上升到80 g/L，脾脏也也有一定的回缩，由原来的163 mm×60 mm到现在的150 mm×50 mm。由此可见依折麦布在治疗由STSL引起的溶血性贫血及血小板减少是有一定的效果的。

## References

[1] Bhattacharyya AK, Connor WE (1974). Beta-sitosterolemia and xanthomatosis. A newly described lipid storage disease in two sisters[J]. J Clin Invest.

[2] Tada H, Kojima N, Takamura M (2022). Sitosterolemia[J]. Adv Clin Chem.

[3] Tada H, Nomura A, Ogura M (2021). Diagnosis and Management of Sitosterolemia 2021[J]. J Atheroscler Thromb.

[4] 张 军, 陈 秋莉, 郭 松 (2022). 以黄色瘤就诊的谷固醇血症患儿临床特征[J]. 中山大学学报(医学科学版).

[5] Rees DC, Iolascon A, Carella M (2005). Stomatocytic haemolysis and macrothrombocytopenia (Mediterranean stomatocytosis/macrothrombocytopenia) is the haematological presentation of phytosterolaemia[J]. Br J Haematol.

[6] Wang G, Cao L, Wang Z (2012). Macrothrombocytopenia/stomatocytosis specially associated with phytosterolemia[J]. Clin Appl Thromb Hemost.

[7] Bastida JM, Girós ML, Benito R (2019). Sitosterolemia: Diagnosis, Metabolic and Hematological Abnormalities, Cardiovascular Disease and Management[J]. Curr Med Chem.

[8] Zhou Z, Su X, Cai Y (2022). Features of chinese patients with sitosterolemia[J]. Lipids Health Dis.

[9] Wang J, Mitsche MA, Lütjohann D (2015). Relative roles of ABCG5/ABCG8 in liver and intestine[J]. J Lipid Res.

[10] Vrins C, Vink E, Vandenberghe KE (2007). The sterol transporting heterodimer ABCG5/ABCG8 requires bile salts to mediate cholesterol efflux[J]. FEBS Lett.

[11] 王 勇, 韩 天权, 张 圣道 (2006). ABCG5和ABCG8与胆固醇代谢关系的研究进展[J]. 国际外科学杂志.

[12] Othman RA, Myrie SB, Jones PJ (2013). Non-cholesterol sterols and cholesterol metabolism in sitosterolemia[J]. Atherosclerosis.

[13] Chan J, Vandeberg JL (2012). Hepatobiliary transport in health and disease[J]. Clin Lipidol.

[14] Yoo EG (2016). Sitosterolemia: a review and update of pathophysiology, clinical spectrum, diagnosis, and management[J]. Ann Pediatr Endocrinol Metab.

[15] Rocha VZ, Tada MT, Chacra A (2023). Update on Sitosterolemia and Atherosclerosis[J]. Curr Atheroscler Rep.

[16] Niu DM, Chong KW, Hsu JH (2010). Clinical observations, molecular genetic analysis, and treatment of sitosterolemia in infants and children[J]. J Inherit Metab Dis.

[17] Kidambi S, Patel SB (2008). Sitosterolaemia: pathophysiology, clinical presentation and laboratory diagnosis[J]. J Clin Pathol.

[18] 苏 雁华 (2007). 伴有红细胞和血小板异常的植物固醇血症临床及发病机制的研究[D].

[19] Kanaji T, Kanaji S, Montgomery RR (2013). Platelet hyperreactivity explains the bleeding abnormality and macrothrombocytopenia in a murine model of sitosterolemia[J]. Blood.

[20] Wang Z, Cao L, Su Y (2014). Specific macrothrombocytopenia/hemolytic anemia associated with sitosterolemia[J]. Am J Hematol.

[21] Zheng J, Ma J, Wu RH (2019). Unusual presentations of sitosterolemia limited to hematological abnormalities: A report of four cases presenting with stomatocytic anemia and thrombocytopenia with macrothrombocytes[J]. Am J Hematol.

[22] 杨 雅景, 薛 梅, 赵 苗苗 (2022). 以血小板减少为首发临床表现的植物固醇血症1例并文献复习[J]. 临床血液学杂志.

[23] Bastida JM, Benito R, Janusz K (2017). Two novel variants of the ABCG5 gene cause xanthelasmas and macrothrombocytopenia: a brief review of hematologic abnormalities of sitosterolemia[J]. J Thromb Haemost.

[24] 陈 梅雪, 宋 元宗, 张 妮 (2021). 1例谷固醇血症患儿的护理[J]. 中华护理杂志.

